# Showering Barriers and Interaction Preferences of Older Adults with Mobility Disabilities for a Robotic Showerhead

**DOI:** 10.3390/healthcare14193149

**Published:** 2026-09-22

**Authors:** Pallabi Bhowmick, Kainat Shahid, Zhiyu Ren, Girish Krishnan, Wendy A. Rogers, Ian M. Rice

**Affiliations:** 1 Department of Health and Kinesiology, College of Applied Health Sciences, University of Illinois Urbana-Champaign, Champaign, IL 61820, USA; kshahid2@illinois.edu (K.S.); wendyr@illinois.edu (W.A.R.); ianrice@illinois.edu (I.M.R.); 2 Department of Industrial and Systems Engineering, The Grainger College of Engineering, University of Illinois Urbana-Champaign, Champaign, IL 61820, USA; zhiyur2@illinois.edu (Z.R.); gkrishna@illinois.edu (G.K.)

**Keywords:** aging, mobility disability, showering, bathing, assistive technology, qualitative research, robotic showerhead

## Abstract

Bathing and showering are among the most challenging daily activities for individuals with long-term mobility impairments, requiring substantial physical effort, coordination, and environmental support. **Background/Objectives:** This study aimed to understand showering challenges faced by older adults with long-term mobility disabilities who used wheeled mobility devices. We evaluated user and caregiver perspectives on three Soft Robotic Showerhead (SoRoSh) concepts: Elongation, Swing, and Two Showerhead designs. We explored preferred interaction modes (user-guided, voice-controlled, gesture-controlled), desired features, and perceptions of how such technologies could improve independence, safety, and usability. **Methods:** We conducted semi-structured interviews with 12 participants: 6 older adults aged 63–77 (M = 72.83; SD = 5.03) with at least 10 years of lower-body mobility disabilities, and 6 subject matter experts aged 30–78 (M = 60.83; SD = 16.82), including professional caregivers and family care partners. Participants discussed current routines, unmet needs, and provided feedback on the concepts and interaction modes. Data were thematically analyzed. **Results:** Participants reported challenges such as difficulty lifting arms, reaching body areas, handling showerheads, maintaining balance, and using unstable shower chairs. Voice control was strongly preferred due to low physical effort, whereas gesture control was viewed as impractical. Manual control was valued for familiarity, with many favoring hybrid approaches. Desired features included integrated controls, joystick input, adjustable spray modes, wake-word activation, and streamlined design. Participants believed these technologies could enhance independence, safety, and emotional well-being. **Conclusions:** Adaptive showering technologies should prioritize accessibility, intuitive multimodal control, customization, safety, and privacy to support independence and quality of life.

## 1. Introduction

Bathing and showering are essential self-care activities that directly affect health, independence, dignity, and quality of life among older adults, particularly those aging with chronic conditions and long-term mobility disabilities [1,2]. Although often viewed as routine hygiene activities, showering requires balance, strength, and coordination for safe movement within a wet and slippery environment [1,3,4]. For older adults with long-term mobility impairments, these demands may exceed physical capacity, leading to increased caregiver dependence, avoidance of showering, fear of falling, and further functional decline [4,5]. Bathing disability is also associated with healthcare utilization and long-term nursing home placement [6]. Thus, showering is not simply a personal-care task; it is connected to safety, hygiene, autonomy, caregiver support, and the ability to age in place.

Showering is often one of the first activities of daily living to become difficult with aging and disability because it requires interaction between the person, the bathroom environment, and the bathing task itself [1]. Older adults with mobility impairments may experience difficulty with transfers, balance, reaching, repositioning, washing, drying, and safely entering and exiting the shower [1,3,7]. Poorly configured bathrooms or inadequate supports can further increase fall risk, fear of falling, caregiver dependence, and shower avoidance [4,8,9]. These challenges affect caregivers by adding physical and emotional demands to an already intimate activity [2,9]. Although home modifications and bathing supports can improve safety and independence, their effectiveness depends on how well they match users’ abilities, routines, preferences, and care needs [3,10].

Beyond physical safety, showering is closely tied to dignity, privacy, emotional well-being, and perceived control. Needing assistance with such an intimate activity may create embarrassment, vulnerability, loss of autonomy, and discomfort with others entering one’s personal space [2,11]. Bathing adaptations can affect not only safety but also how clean, independent, confident, and in control people feel [2]. Professional care providers similarly describe bathing assistance as challenging because it combines safety risks, limited space, physical handling, multitasking, and intimate personal care [9]. Bathroom design must therefore support both client autonomy and a safe, sustainable work environment for caregivers [12]. Showering interventions should consequently preserve privacy and self-direction while reducing physical risk and caregiver burden.

Existing bathing supports include grab bars, shower chairs, transfer benches, handheld showerheads, level-access showers, non-slip flooring, bath lifts, and other assistive products intended to improve safety, access, and independence [1,13]. When appropriately matched to users and their environments, such supports can improve shower and bathing transfers [3]. Their effectiveness, however, depends on design and placement; for example, King and Novak [14] found greater instability during bathing transfers and demonstrated that some grab-bar and wall-touch configurations supported safer transfer control than others. Access also remains uneven. Lam et al. [15] reported that 42% of older adults who needed bathing or toileting equipment lacked access to it, with barriers including cost, awareness, professional guidance, environmental constraints, and difficulty matching products to individual needs [1,15].

User-centered research has examined decisions around bathroom assistive technology. Guay et al. [16] developed Hygiene 2.0 to help older adults and caregivers select bathroom-assistive technologies with consideration for safety, autonomy, and usability. However, such work focused primarily on selecting existing aids rather than designing adaptive technologies that actively assist with water delivery, showerhead positioning, or reaching difficult body regions.

More broadly, current bathing supports, including mobile shower commodes, grab bars, shower chairs, transfer aids, and environmental adaptations, primarily address access, seated support, transfer safety, and stability [1,2,7]. Because these solutions are generally static or transfer-oriented, they may provide limited support during washing and rinsing, when users must reach difficult body areas, reposition water flow, manage fatigue, and maintain balance. Existing supports can become barriers when poorly positioned, difficult to use, or mismatched with users’ abilities and routines [1,7]. Findings from the Aging Concerns, Challenges, and Everyday Solution Strategies (ACCESS) project showed that technologies intended to compensate for activity limitations introduced new challenges when their design did not meet users’ needs [5].

Research on technology and aging with disability has provided a foundation for addressing these limitations. The TechSAge Technology Intervention Model conceptualized technology as a means of bridging the gap between intrinsic capabilities and functional abilities among adults aging with long-term disabilities [17]. This perspective is particularly relevant to showering, where performance is shaped by changing physical capacity, environmental barriers, safety risks, assistive supports, and personal preferences. Older adults do adopt new technologies when they are meaningful, usable, and compatible with their needs and contexts [18]. Accordingly, showering technologies should not simply automate hygiene tasks but support safety, comfort, independence, privacy, and control.

Recent work has explored bathing adaptations, mechatronic bathing systems, robotic bathing, and smart shower technologies, yet these approaches have not fully addressed the everyday showering needs of older adults with long-term mobility disabilities. Golding-Day and Whitehead [19] found that home bathing adaptations influenced confidence, independence, and caregiving experiences beyond immediate safety benefits, but focused on environmental adaptations rather than active assistance during washing and rinsing. Bezerra et al. [20] developed Bath-Ambience, a portable mechatronic system for bathing bedridden individuals, demonstrating the feasibility of mechatronic bathing assistance but addressing bed-based care rather than partially independent showering.

Robotic systems have been developed for more intensive bathing assistance. Zlatintsi et al. [21] presented I-SUPPORT, which integrated environmental perception, human–robot interaction, a motorized chair, and robotic assistance for bathing tasks. Although this work established bathing as an application area for assistive robotics, large robotic platforms may be difficult to integrate into ordinary home bathrooms because of space, installation, maintenance, privacy, and cost. More targeted approaches, such as adaptive showerheads, may provide physical assistance without requiring replacement of the entire bathing environment.

The social and ethical implications of robotic showering are important considerations as well, particularly given the intimate nature of showering and the potential use of sensing and monitoring technologies. Klein and Schlömer [22] emphasized dignity, autonomy, privacy, and acceptance in relation to robotic shower systems. Related research on smart-home sensing has similarly highlighted privacy and obtrusiveness as important considerations when monitoring technologies are introduced into older adults’ homes [23]. These concerns are directly relevant to smart shower systems such as that proposed by Hossain and Abdelgawad [24], which incorporates environmental sensing, fall prediction, and IoT-based caregiver monitoring. Although such features may support safety, they also raise questions about privacy, autonomy, consent, and acceptable levels of caregiver oversight during an intimate activity. Thus, broad ethical considerations must be translated into specific design decisions concerning sensing, user control, data handling, caregiver involvement, and the physical assistance provided by the system.

Interaction research has begun to examine alternative controls for smart shower environments. Chen et al. [25], for example, investigated user-defined foot gestures for eyes-free interaction. Such approaches recognize that users’ hands may be wet, occupied, or needed for balance; however, foot gestures may not be feasible for people with lower-limb weakness, spasticity, pain, poor balance, or wheelchair use. This illustrates the importance of considering mobility disability when selecting interaction modalities for assistive shower technologies.

A recent review by Li et al. [26] further identified bathing assistive devices and robots as an emerging research area and categorized technologies according to bathing methods, modes, post-bath care, structural design, motion control, and information intelligence. However, much of this research has emphasized device structure, automation, and technical function, with comparatively less attention to users’ lived showering experiences, difficult-to-reach body regions, preferred showerhead movements, acceptable sensing, and how control should be shared among users, caregivers, and technology.

Taken together, prior work has addressed home bathing adaptations, mechatronic bathing systems, robotic platforms, ethical issues, smart shower sensing, monitoring, and alternative interaction techniques [14,16,19,20,21,22,24,25,26]. However, comparatively little research has examined adaptive showerhead technologies that directly support water delivery, body-region access, repositioning, user control, caregiver involvement, privacy, and dignity during everyday showering. Showering independence depends not only on safely entering the shower but also on being able to wash and rinse effectively, comfortably, and with a sense of control.

To address these gaps, the present study assessed the needs and preferences of older adults aging with long-term mobility impairments and subject matter experts (SMEs) to inform design requirements for future bathing and self-care technologies. Specifically, we explored showering experiences, barriers, facilitators, safety concerns, interaction preferences, and desired design features for an innovative showerhead system. By examining showering as an essential self-care activity linked to healthcare, our goal was to inform technologies that support safe, independent, dignified, and acceptable self-care for older adults with long-term mobility disabilities.

## 2. Materials and Methods

### 2.1. Recruitment

We recruited two participant groups: older adults with long-term mobility impairments who used wheeled mobility devices and SMEs who included family care partners and professional caregivers. This sampling approach was designed to obtain complementary perspectives from individuals with lived experience and individuals with caregiving expertise related to mobility support and bathing assistance. A dyadic design was not used because the objective of the study was not to examine care-recipient–caregiver relationship dynamics within matched pairs, but rather to capture broader, independent perspectives from each stakeholder group regarding showering challenges, unmet needs, and robotic showerhead design preferences. This approach allowed for participants to discuss sensitive showering-related experiences openly without the influence of a paired caregiver or care recipient.

Recruitment materials included a brief study description, eligibility criteria, study procedures, expected time commitment, and contact information for the research team. Older adults were recruited through community organizations, clinical and rehabilitation-related networks, senior centers, registries, social media postings, flyers, and referral-based networks. SMEs were recruited through professional networks, caregiver organizations, and referrals. Individuals who expressed interest contacted the research team and completed an eligibility screening process prior to enrollment.

#### 2.1.1. Inclusion and Exclusion Criteria

Older adults were eligible to participate if they were 60 years of age or older; self-reported long-term mobility impairment (for at least 10 years); used a wheeled mobility device (e.g., wheelchair or scooter) for at least 40 h per week; needed to shower in a seated position; had access to a computer and internet connection; and were able to read information presented on a computer screen. Individuals who did not meet these criteria or were unable to participate in an online interview were excluded.

SMEs were eligible if they were family care partners or professional caregivers with at least one year of experience supporting wheelchair users. Individuals without relevant caregiving experience with wheelchair users or bathing-related support needs were excluded.

#### 2.1.2. Screening

We conducted screening through REDCap (version 17.4.6) [27,28] and included contact information, eligibility confirmation, and verification of Zoom access. Cognitive screening was completed only for older adults using the Telephone Interview for Cognitive Status–Modified (TICS-M) [29], with a required score of 31 or higher. Eligible participants were scheduled for an interview.

#### 2.1.3. Data Collection

Data were collected through individual semi-structured Zoom interviews lasting approximately 1.5 to 2 h. Older adults completed a background questionnaire, showering challenges questionnaire, and video-based design feedback interview. SMEs completed demographic screening, caregiving history and experience, showering support questions, and video-based design feedback. Given the exploratory and design-oriented aims of the study, sample adequacy was considered in relation to specificity of the participant groups, relevance of participants’ experience to the study questions, and richness of the interview data, consistent with the concept of information power [30].

### 2.2. Participant Demographics

Demographic and background characteristics were collected using the TechSAge background questionnaire [31] and screening materials for older adults and SMEs respectively. Variables included age, gender, race, disability status, duration of disability, and caregiving experience. The final sample included 12 participants: 6 older adults and 6 SMEs.

Older adult participants ranged in age from 63 to 77 years (M = 72.83, SD = 5.04). As shown in Table 1, the older adult sample included both male and female participants and represented participants who self-identified as White or Black/African American. Participants reported long-term mobility-related disabilities, including post-polio syndrome, Ehlers–Danlos syndrome, and multiple sclerosis, with disability durations ranging from 15 to 75 years.

SMEs ranged in age from 30 to 78 years (M = 60.83, SD = 16.82). As shown in Table 2, the SME sample included caregivers with varied racial backgrounds and caregiving experience ranging from 3 to approximately 30 years. These participants provided perspectives based on professional or family caregiving experience with individuals with mobility limitations. All SME participants had direct experience assisting with bathing and/or showering throughout their caregiving experience. Professional caregivers and family care partners were intentionally analyzed together because the purpose of the SME sample was not to compare caregiving roles, but rather to capture perspectives from individuals with direct experience supporting individuals with mobility limitations during bathing and showering. Both groups were included as SMEs based on this shared experiential knowledge.

### 2.3. Study Design and Procedure

#### 2.3.1. Study Setup and Overview

The study was approved by the Institutional Review Board (IRB24-2162) and conducted entirely online via Zoom. Using a single-session, semi-structured interview design, we explored bathing/showering challenges, support needs, and perceptions of a robotic showerhead. Each session lasted approximately 1.5 to 2 h.

Before the session, participants received study information, the consent form, and Zoom instructions by email. Screening, scheduling, eligibility verification, and cognitive screening, when applicable, were completed in advance. Prior to each session, the research team confirmed that REDCap, Zoom, audio, video, screen sharing, recording, and transcription functions were working properly. The study flow was adapted for the two participant groups as shown in Figure 1 for older adults and Figure 2 for SMEs. The semi-structured interviews were conducted by the lead researcher who had prior training and experience in qualitative interviewing and research related to human factors, aging, disability, and/or assistive technology, while a second team member served as notetaker to document session flow, participant comments, and technical issues. Neither the interviewer nor the note-taker had any prior personal relationship with any of the participants.

#### 2.3.2. Robotic Showerhead Concepts and Interaction Modes

Three robotic showerhead concepts were evaluated: elongation, swing, and two showerhead, as shown in Figure 3. The Elongation concept depicted a variable-length robotic arm designed to extend the showerhead toward hard-to-reach body regions, particularly posterior areas such as the back. The Swing concept depicted an articulated robotic arm that moved the showerhead along a curved trajectory to provide broad coverage around the seated user. The Two Showerhead concept depicted a distributed system with separate showerheads for front and back washing, intended to improve access to posterior body regions and reduce the need for one showerhead to cross the body.

Each concept was demonstrated using three interaction modes: user-guided, voice-controlled, and gesture-controlled. In the user-guided mode, participants saw a direct physical-control approach in which the showerhead or robotic arm could be manually repositioned and then remain in place after release, reducing the need for sustained holding. In the voice-controlled mode, users operated the showerhead through spoken commands, such as directional or start/stop commands, to support hands-free control. In the gesture-controlled mode, users directed the showerhead through noncontact hand or arm movements using vision-based or depth-sensing input.

The order of presentation of the three showerhead concepts was counterbalanced across participants to distribute potential presentation-order effects and minimize the possibility that evaluations of a particular concept were systematically influenced by its position in the sequence, including primacy or recency effects [32,33], as shown in Table 3. Within each concept, however, the interaction modes were intentionally presented in a consistent sequence from user-guided to voice-controlled to gesture-controlled. This progression reflected increasing interaction novelty and was intended to scaffold participants’ understanding; namely, user-guided interaction most closely resembled conventional showerhead use, voice interaction was expected to be relatively familiar through commercially available voice assistants, and gesture interaction represented the least familiar modality.

Participants were asked to identify their preferred concept and interaction mode, describe desired physical and functional features, comment on sensors and cameras, evaluate perceived effects on independence, and discuss willingness to use or pay for the system. Participants were asked to describe their ideal robotic showerhead design, which provided additional insight into unmet needs and future design opportunities.

### 2.4. Data Recording, Transcription, and Anonymization

Interviews were audio- and video-recorded and stored in secure project folders designated for protected health information, accessible only to authorized research team members. Recordings were reviewed only in private settings, with headphones used during playback, and were transferred to secure storage after each session for transcription.

Transcription used a combined automated and manual verification process. Audio recordings were first processed in Otter.ai (version 3.136.0) to generate draft transcripts. A four-member team then reviewed each transcript against the recording to correct errors and ensure accuracy. Transcripts were prepared verbatim, preserving natural speech patterns, pauses, interjections, and relevant nonverbal expressions. Speaker labels, timestamps, formatting, and annotations for unclear audio were standardized.

All transcripts were de-identified before analysis by removing or replacing names, locations, organizations, health-related identifiers, and other personally identifiable information with descriptive placeholders. The lead team member reviewed each transcript for de-identification, formatting, and quality. Final transcripts were labeled with unique participant IDs and stored separately from identifying information. Transcription logs documented assignments, completion status, quality checks, and relevant notes.

### 2.5. Qualitative Analysis

We used a team-based codebook thematic analysis using an inductive coding strategy outlined by Braun et al. (2019) [34] and Braun and Clarke (2021) [35] to identify patterns in participants’ experiences and perspectives. The codes were initially generated from the data rather than established a priori. Rather than applying a predetermined coding framework, codes were developed from participants’ accounts and iteratively organized into broader categories and themes relevant to the study aims. The analysis was conducted by three researchers trained in qualitative research. Researchers independently reviewed and coded assigned transcripts using an inductive coding strategy, while the lead researcher coordinated development and refinement of the shared coding framework, theme development, and organization of the final analytic narrative.

Researchers first familiarized themselves with the data by reading and rereading the transcripts and consulting recordings when clarification was needed. Initial coding was conducted through detailed review of the transcripts, with codes capturing experiences related to showering challenges, caregiving demands, technology perceptions, usability concerns, design preferences, and implementation considerations. Codes were documented in a shared Excel-based coding framework and iteratively refined as analysis progressed. This process included clarifying code definitions, consolidating conceptually overlapping codes, and adding new codes when relevant concepts were identified.

Related codes were subsequently grouped into preliminary thematic categories based on conceptual similarity. These categories were reviewed in relation to the coded excerpts, full transcripts, and study aims to assess their coherence, distinctiveness, and relevance. Findings were examined across older adults and SMEs to identify areas of convergence as well as differences in perspectives between the two groups.

Regular analytic meetings were held throughout the process to discuss coding decisions, clarify interpretations, and refine the coding framework and emerging themes. Differences in coding or interpretation were used to identify areas requiring further clarification and were discussed collaboratively by the research team. Through this iterative process, themes were refined, defined, and organized into the final analytic structure. Representative quotations were then selected to illustrate the range and depth of participants’ perspectives within each theme.

## 3. Results

Analysis identified seven cross-cutting themes reflecting participants’ showering experiences and their perceptions of the proposed robotic showerhead concepts: (1) changing functional abilities reshaped showering routines and autonomy; (2) stable and flexible positioning reduced physical demands; (3) concept preferences reflected tradeoffs between familiarity, reach, and complexity; (4) interaction preferences depended on functional fit and multimodal control; (5) learning demands and command interpretation shaped anticipated usability; (6) personalization, privacy, and shared use influenced acceptability; and (7) practical feasibility extended beyond interaction usability. Older adult and SME perspectives frequently converged, although SMEs additionally emphasized caregiving, safety, and implementation considerations.

### 3.1. Changing Functional Abilities Reshaped Showering Routines and Autonomy

Older adult participants described how changes in mobility, upper-body strength, and the availability of environmental supports altered their established showering routines. Activities that had previously been manageable, such as lifting an arm, using grab bars, or handling soap, became increasingly difficult as functional abilities changed. One participant explained how declining arm function affected both positioning and product use during showering.

*“I used to be able to raise my specifically, my right arm up so that I could I had. We used to have a shower seat that folded down and a grab bar that folded in front of me, so I had something in front of me, and I had the wall behind me. I was on a padded seat, um, I can’t do that anymore. I could use the back wall, you know, the corner to rest, you know, press my elbow against, keep my arm up. I can’t do that anymore. That’s change. That’s part of the reason I stopped using bars of soap.”*

Some participants reported showering less frequently as mobility limitations and inadequate accommodations made the activity more physically demanding. Rather than following a fixed routine, participants sometimes based the decision to shower on necessity, anticipated activity, or whether the effort involved could be justified.

*“I’m down to two showers a week.”*

*“I don’t shower quite as often if I’m not out running around.”*

Within this context, independence emerged as more than the ability to complete a physical task. Older adults associated independence with enjoyment, autonomy, and retaining control over everyday activities.

*“I think it made my independence more enjoyable.”*

*“I don’t want to give up control on wheels. I can push if necessary.”*

SMEs similarly described the emotional consequences of declining independence. They observed frustration when care recipients perceived that family members or caregivers were monitoring them too closely.

*“With my mom, she got a little snippy about us checking in or hovering.* *”*

*“Watching her be frustrated wanting to be more independent is what is most challenging.* *”*

Participants consequently evaluated the robotic showerhead partly in terms of whether it could allow users to maintain control over portions of the showering process. Older adult participants anticipated that the technology could extend the amount of time they remained able to shower independently:

*“physically, I think it would extend the period of time in which I could shower without requiring someone else to be in there bathing me, which gives me more independence, more self control.”*

*“Physically, I think that something like this would be likely to give me a more complete and more thorough bathing, showering experience.”*

SMEs similarly perceived potential for the showerhead to reduce direct caregiver involvement, help care recipients regain a degree of control, improve independence, not feel like a burden, and provide a sense of empowerment, particularly during private aspects of bathing.

*“So, it might potentially help with them feeling a sense of independence around being able to watch their private area on their own, some of the things that they don’t want the caregivers to do.”*

*“They don’t feel like, you know, somebody is doing this for them, like they’re dependent on a person. We don’t want them to feel like they were little kids. It takes that feeling away, which is not easy.”*

*“Gives them some independence back […] they don’t feel like they’re an affordable burden and you don’t feel strange.”*

*“I think in our personal situation there, there are times when this would be very helpful and empower the care recipient.”*

At the same time, SMEs emphasized that increased independence did not necessarily mean eliminating caregiver involvement. Assistance might still be necessary for transfers, fall prevention, or other aspects of showering.

*“Balance between letting them be independent and then also the battles of them not following what they needed to do to be safe.”*

*“We didn’t want them to feel like they were little kids. You know, we’re hovering over you. And we also wanted them to understand we were there if they needed something.”*

Participants and SMEs also emphasized that the potential effect on independence would vary according to users’ existing abilities and shower setups.

*“The question is dependent upon the mobility of the person themselves.”*

*“He has always been quite independent, I don’t know if he would find it worth his time.”*

Together, these accounts positioned independence as conditional and multidimensional, involving functional ability, perceived control, privacy, emotional well-being, and the nature of caregiver involvement.

### 3.2. Stable and Flexible Positioning Reduced Physical Demands

Across the concepts, participants consistently valued the ability to position the showerhead without continuously holding it. Conventional handheld showerheads required participants to grasp, aim, and manipulate the device while simultaneously washing or maintaining balance. For individuals with limited strength or unilateral impairment, this could leave few available options for completing other showering tasks. Older adult participants described the value of being able to reposition the showerhead and then release it, while also connecting hands-free positioning to physical safety because both hands could remain available for stabilization.

*“Well, I think it would be really useful to be able to move the showerhead around without having to hold on to it and have it stay in place despite the water pressure. That is a very interesting concept, because with with even a handheld shower, you have to hold it, you have to direct it, and if you’re holding it, then at best, you have only one hand, if you can use your second hand, which I can’t use it very much to wash. So being able to have your hands free for using, you know, the soap, the body wash, whatever you use, that is very useful, I think.”*

*“If you want it directed away from you for a minute while you’re soaping up, and then you want to be able to pull it back. And you want to be able to, like, rinse your hand before you grab it to pull it back into position. That’s very handy.”*

*“I think it would add to our safety in the shower, not to have to hold it all the time, to have the option to hold it, move it, but then take our hand. We’ve got two hands to hold us in position, and, you know, make sure we’re safe and secure in our chair.”*

SMEs similarly emphasized the value of hands-free positioning.

*“I think not having to hold onto it while using both your hands to wash your face, wash your body, wash your hair, I think that’s a useful feature* *.”*

*“I like that they can stay in place so someone can sort of stay warm in the shower instead of freezing.”*

Participants also valued the ability to move the showerhead across different body areas while remaining seated. Older adults described reach as particularly important.

*“It’s flexible enough that it reaches the back.”*

*“You don’t have to lift it over your body and then aim it down.”*

*“The fact that I can move it without standing up.”*

Those participants with limited ability to retrieve the showerhead from the shower floor appreciated that this showerhead will not fall or drop off on the floor. They also appreciated the reduced likelihood of dropping it due to wet, soapy hands.

*“I think mostly that you don’t have to worry about dropping it because soapy hands and wet hands and poor grasp, that that all that would be nice.”*

*“I think it’s good that it can move around and also stay in the last place you decided to leave it and it, I mean, it will not fall on the floor and bounce off your foot. That’s very good.”*

However, older adults and SMEs alike raised concern over the effort needed in moving the showerhead.

*“I’m just worried that it’s going to be too hard to move.”*

*“I guess what I would like to know is how difficult it is to move?”*

*“I’m not sure how easy dragging it to the back will be, particularly for elderly.”*

Thus, stable positioning and ease of movement were closely related but distinct considerations. Participants valued a showerhead that remained securely where placed while also emphasizing that reaching, repositioning, and manipulating the device needed to remain within users’ functional capabilities.

### 3.3. Concept Preferences Reflected Tradeoffs Between Familiarity, Reach, and Complexity

Participants’ preferences among the three showerhead concepts reflected differences in how familiar, physically accessible, and complex each design appeared. The Elongation concept was frequently viewed favorably because it retained a familiar front-facing configuration while extending the showerhead’s reach and allowing it to remain positioned where needed. Participants perceived this design as relatively straightforward and similar to conventional showering arrangements. As shown in Table 4, the Elongation concept was frequently viewed favorably because it retained a familiar front-facing showerhead configuration while extending its reach and allowing the showerhead to remain positioned where needed.

*“Elongation has one fixed point. It’s easily more adaptable to the majority of homes and apartments and living facilities that are constructed.”*

The Swing concept was valued particularly for its range of movement and ability to reach different areas of the body. Some participants viewed its movement as comparatively easy to manipulate and noted its potential to provide coverage across the front and back of the body.

*“The swing one because it’s less to manipulate. It’s easier.”*

*“It (Swing concept) has to get to be able to do the front and the back, and that’s a plus.”*

Responses to the Two Showerhead concept were more mixed. There were confusions around which showerhead would be active at a given time and the need for separate commands to activate or control each one. While some participants valued the possibility of increased body coverage, others perceived the second showerhead as unnecessary or more complicated than a single movable showerhead. One participant explained:

*“I think they can do it all with one. I think this was overkill and too expensive for enough people to afford.”*

A couple of participants preferred a hybrid configuration that combined characteristics of the Swing and Elongation concepts rather than selecting either concept independently. Their comments suggested that they saw the two movement mechanisms as complementary: the Swing concept offered lateral movement around the body, while Elongation could extend the showerhead vertically or toward the user to increase reach and positioning flexibility. Availability of multiple concept options as a form of choice could itself contribute to a sense of control.

*“I like the swing showerhead concept. But as I mentioned, it might be, but it might be easier if it elongates, and they could swing it too.”*

*“Okay, because it’s kind of the same thing as the swing. It’s just you can elongate it so you could go high, you could go low, but you could always move it around.”*

Overall, concept preferences reflected a balance between expanded reach and coverage on one hand and familiarity, ease of manipulation, and perceived complexity on the other. Participants generally responded more favorably to concepts that extended functional reach while remaining close to familiar showerhead configurations.

### 3.4. Interaction Preferences Depended on Functional Fit and Multimodal Control

Participants evaluated user-guided, voice-controlled, and gesture-controlled interaction in relation to users’ remaining motor, communication, sensory, and cognitive abilities. Rather than identifying one interaction mode as universally appropriate, they described circumstances in which different modes might become more or less useful. A hybrid approach combining both voice-controlled and user-guided mode emerged as the preferred choice as shown in Table 3.

Voice control was frequently viewed as valuable when physical reach or upper-extremity movement was limited. An older adult explained:

*“If there’s something that I couldn’t reach in the back, it would have to be voice controlled…and the rest of it could be hand controlled.”*

SMEs also perceived voice interaction as potentially less physically demanding.

*“For my husband, specifically with his MS, that the heat of the shower weakens you, and if, if he already has one sided weakness, the hand controls, voice control would be better.”*

However, participants recognized that voice interaction would not be accessible to everyone.

*“Some people have trouble with voice and they don’t have much voice left there, especially the ones that had the lung trouble, they may not have enough voice to get it moving, but their hands might be able to move.”*

*“if someone cannot speak or be clear enough the gestures would be good.”*

*“When you talk about people with disabilities, if they have cerebral palsy, or anything else, if we’re reliant upon the verbal command, are they able to articulate? Because otherwise the verbal part of it is moot.”*

Gesture interaction offered an alternative for some users but introduced different physical demands. Participants with limited arm movement questioned whether they could reliably perform the required movements. Additionally, involuntary movement during showering presented another concern, while one participant even noted potential confusion with directional commands.

*“But again, if someone has some limitations. For example, I can use my right arm maybe 25% as well as someone who’s not disabled. My left arm, it’s at most five to 10% so raising my left arm will not work.”*

*“I also think that if someone, for example, had either cerebral palsy or another disability in which there’s some spasms. I’m not sure if there’s an easy way to train this to know what movement is meant for the shower.”*

*“What if you’re dealing with a dyslexic or a person on the spectrum, lots of the people on the spectrum, be they young or old, cannot tell their left from the right. And they go, Oh, do this. And they’re like, What the hell does that mean?”*

Participants repeatedly emphasized considerable heterogeneity among people with mobility disabilities. Some participants perceived greater value for people with substantial upper-extremity limitations than for those who could already use conventional shower fixtures. Participants noted that combinations of motor, communication, sensory, and cognitive limitations could make particular interaction modes difficult.

*“But among the polio group, some people have good arms. Some people have trouble with voice, like that. There’s a wide variety. And then among multiple sclerosis and all these other things, people are getting cerebral palsy.”*

*“So there will be some people who present unique things that I don’t know how much this can be designed with the kind of adaptability to address things like spasticity, what its functionality is for understanding what somebody says.”*

*“For me, this would not be worth it but somebody that has no choice, that really can’t use their hands at all, if somebody’s quadriplegic, I could see where this would be nice”*

*“If you can’t do movement and you can’t talk you would be at a point where you can’t use and of these.”*

Participants imagined switching modes according to the showering task rather than selecting one mode for the entire interaction.

*“Have elongate and then, and then you use it, and then maybe use the voice for the shampooing and rinsing.”*

Overall, perceptions of interaction modes were closely tied to functional fit. Participants’ accounts emphasized variation in strength, range of motion, speech, involuntary movement, vision, cognition, and prior ability rather than a single interaction preference applicable to all users.

### 3.5. Learning Demands and Command Interpretation Shaped Anticipated Usability

Besides mobility limitations shaping usage, both older adults and SMEs anticipated an initial period of adjustment when learning to operate the robotic showerhead. Participants expected users to become familiar with available commands, understand how the system interpreted those commands, and develop confidence in predicting its response.

*“A lot to keep track of, but with practice, I can see how you could do that.”*

*“I think it would take a little bit of getting used to so that you make sure that you have your voice telling you exactly what you wanted to do.”*

*“It’s just the learning curve for the commands.”*

*“if we put a waterproof directions that would that would help her so that if she was sitting there, she would know what commands.”*

*“…once you train yourself on what the commands are [voice-controlled mode] and how it reacts to the commands that could accomplish a lot of things.”*

Participants described differences in the anticipated learning burden associated with different interaction modes. Voice was generally perceived as more familiar because speaking was already an established mode of communication. Gesture control, by comparison, required participants to learn and remember mappings adding to cognitive load and frustration, while some participants questioned whether gesture recognition could distinguish intentional from involuntary movement.

*“With the gesture part I would have to get used to that. Since I talk all the time, I think it’d be easy for me to tell it on or off and right and left.”*

*“but I think it’s [gesture-controlled mode] much more limited in value compared to the voice one, in part, because you really have to learn what gesture does what.”*

*“it’s [gesture-controlled mode] more complicated to try and figure out how to use so it might have a longer learning curve.”*

*“I’m not sure if there’s an easy way to train this to know what movement is meant for the shower, as opposed to what is an involuntary kind of movement.”*

*“I have a few friends that some of these things get too technical, and they just don’t want anything to do with it.”*

SMEs also emphasized that learning demands could vary substantially according to cognitive and functional abilities.

*“He was starting to get dementia, so he trying to help him to understand that he would turn the showerhead off and on while he was washing his hair…he didn’t understand how to adjust the temperature so that would frustrate him.”*

*“My dad, he wouldn’t be able to do the voice- or gesture-controlled because of his of his dementia.”*

Together, these findings indicated that anticipated usability was shaped not only by what each mode allowed users to accomplish but also by the effort required to learn, remember, communicate with, and develop trust in the system.

### 3.6. Personalization, Privacy, and Shared Use Influenced Acceptability

Participants evaluated the robotic showerhead as part of a highly personal activity taking place within a shared household environment. Consequently, their perceptions extended beyond basic operation to include customization, aesthetics, accommodation of other users, and privacy. Several older adult participants expressed preferences for wider, rain-showerhead styles, while being able to regulate the type of spray, water pressure intensity, and temperature, for comfort and safety.

*“I think I would like more of a rain shower type.”*

*“I would want a wide shower spray… I would just prefer just a wide flow of water.”*

*“Being able to regulate the water pressure and regulate the type of spray, whether it’s a broad spray or a narrow spray.”*

*“You need adjustment for temperature, some kind of voice commands that will adjust your temperature control.”*

*“I am a huge proponent of those showerhead having a thermostatic shutoff for burn temperatures.”*

Aesthetics also shaped anticipated acceptance. Older adults preferred a device visually compatible with the bathroom.

*“Well, I want it to be stainless steel to match the rest of the features in the shower area.”*

*“And I would want it to be as sleek and modern looking as possible.”*

These concerns became particularly important because the bathroom and shower were generally shared. Older adults repeatedly considered how the proposed system would affect spouses, relatives, and guests.

*“Because I’m not the only one that uses the shower, I’m not sure how my husband would react to that. If it could have setting one for a spouse, say, and setting two for the other person, you know if it’s only, if it’s a shared shower, you want it To be able to share with a guest or something.”*

*“So if you’re talking about it in the home, I mean, consideration ought to be given to that other person who is more likely not not disabled. Or if they are disabled, they may have a very different disability…”*

*“I don’t know how much room this is going to take up in the shower, especially for the other parties who use it* *.”*

Privacy became especially salient when participants considered the possible use of cameras and sensors. Older adults expressed immediate discomfort with visual monitoring and also questioned where data might be transmitted and who could access it.

*“If I get to control access to that, how do I know that I have full control over that?”*

*“Well, I think that would kind of creep me out, frankly, whether you know, I wouldn’t like, I would not like that. There has to be a better, you know, heat sensor or something to sense your, your body shape or whatever. But camera, no.”*

*“I’m not thrilled with cameras. They can be hacked so easy.”*

*“The sensors have to be such that they don’t pick up a motion or action outside of the shower.”*

Together, these findings showed that acceptability was shaped by the showerhead’s fit within both the individual showering experience and the broader household environment, including personal preferences, shared use, aesthetics, and expectations of privacy.

### 3.7. Practical Feasibility Extended Beyond Interaction Usability

Participants’ evaluations of the robotic showerhead were influenced by practical concerns regarding installation, plumbing, water containment, maintenance, and cost. Installation feasibility was a prominent consideration. Participants questioned whether the system could be incorporated into an existing bathroom without substantial renovation, compatibility with existing plumbing, housing constraints, water containment, maintenance requirements, and affordability.

Installation feasibility was a prominent consideration. Participants generally viewed systems requiring fewer plumbing modifications as easier to implement. Consequently, the Elongation concept was received favorably as participants perceived its single-point configuration as comparatively straightforward to integrate into existing bathrooms.

*“The adaptability of implementing the elongated concept, because it can go into existing plumbing without any behind the wall adaptation necessary.”*

Easy integration was assumed to mean less associated cost. Participants frequently viewed simpler concepts more favorably as more financially realistic:

*“In terms of cost, you’d look for the one with the least mechanics.”*

*“Elongation showerhead concept I think that would be the simplest for installation and cost.”*

*“If this can be added to an existing bathroom, then potentially your renovation cost isn’t that great, right?”*

*“It also might be cheaper, because you’re not going to have two lines for plumbing.”*

Similarly, concepts involving multiple showerheads or water sources prompted additional concern.

*“The second arm (Two Showerhead concept) is out of question because you would have to find a way to run a water line to the back of a shower.”*

*“You would have to have piping at the rear end of the shower.”*

*“I can’t imagine that it (Two Showerhead concept) would be worth the expense to have two showerheads.”*

Water containment represented another practical concern. The increased movement of the Swing concept prompted concerns related to directing and containing water within the showering area and whether it could increase the risk of spraying beyond the intended area. Thus, its greater mobility could be perceived as both an advantage (as noted before) and a potential source of complexity.

*“First, what it does is increases the chance of flooding the bathroom floor.”*

*“The motions of that would have to be so refined that you would not get over spray.”*

*“I’m concerned, if I said right or left or front, that it would go too much and start squirting up my bathroom or my wheelchair.”*

Water on the bathroom floor was associated with perceived safety risk:

*“[…] because getting out of the tub on a wet floor is extremely dangerous.”*

Housing context further affected perceptions of feasibility. Participants living in apartments or senior living environments described limited authority to modify the bathroom.

*“I live in a senior living community. I would have to get permission from the management here.”*

SMEs similarly noted that even households with sufficient resources might not have ideal bathroom infrastructure or be willing to undertake renovation.

*“Even this extremely wealthy family I work for right now still doesn’t even have a roll in shower […] I don’t think nobody likes to renovate their shower.”*

Affordability emerged as an important point of concern overall. Participants questioned whether the technology would be affordable for home use.

*“My first thought was expensive. Very few people can afford this.”*

*“Whether or not there’s enough people that can afford to use this to make it worth that.”*

Participants considered insurance coverage and out-of-pocket affordability. Older adult participants suggested that insurance or Medicare coverage could influence whether the technology would be affordable or adopted.

*“How much would I pay for the product or service to have it as a convenience over what I have, I would say, $50. Insurance could cover the rest of it.”*

*“It would be my parents paying for it, if insurance didn’t pay, I’m not sure she would consider it. Tell Medicare to pay for it. That would be nice.”*

SMEs discussed specific price points, copays, and what might be reasonable for users or families.

*“My aunt’s budget would be about $30 copay.”*

*“If they had to pay for it outright, probably $50–$60.”*

*“$50 copay. For a lot of the population would be reasonable.”*

Together, these accounts demonstrated that participants assessed feasibility at multiple levels. Anticipated usability of the interaction itself was only one consideration; participants also evaluated whether the technology could realistically be installed, maintained, afforded, and safely incorporated into existing bathrooms.

## 4. Discussion

This study examined perceptions of older adults with long-term disabilities who use wheeled mobility devices and shower while seated and individuals experienced in providing bathing assistance. We explored their viewpoints about the design, operation, and potential value of three soft robotic showerhead concepts and multiple interaction approaches. Their perspectives demonstrated that the acceptability of such a system depended on more than its ability to move water around the body. Participants evaluated the concepts in relation to whether they could reduce physical demands while preserving control, accommodate heterogeneous abilities, remain understandable and predictable, fit within a shared and private household environment, and be realistically integrated into existing bathrooms. Therefore, the potential of the robotic showerhead to increase independence is substantial but conditional.

The stated considerations pointed toward assistive showering technology as a configurable support that extends users’ capabilities rather than as a fully autonomous replacement for existing showering practices or caregiving support. This interpretation is consistent with prior assistive technology research emphasizing that successful technology use depends on the fit among the technology, the individual’s functional abilities, the activity being supported, and the circumstances in which that activity occurs. This interpretation aligns with person–environment fit research showing that disability-related outcomes in later life are shaped by the relationship between individual capabilities and environmental demands rather than by either, in isolation [36]. Our findings extended this perspective to robotic showering assistance by illustrating how this fit encompasses not only physical accessibility but also interaction modality, familiarity, privacy, household use, and implementation constraints.

### 4.1. Supporting Independence Through Selective Assistance Rather than Full Automation

Independence in showering should not be understood simply as completing the entire activity without assistance. Participants connected independence with retaining control, maintaining privacy, dignity, completing personally manageable portions of the activity, and reducing unnecessary caregiver involvement. At the same time, caregivers may continue to play important roles in transfers, fall prevention, setup, or emergency assistance. Thus, the more relevant design goal may be supported independence, in which technology enables users to perform more of the activity themselves while assistance remains available where necessary.

This distinction is particularly important for intimate activities such as showering. Reducing hands-on assistance may have value even when it does not eliminate caregiving altogether because it can change which parts of the activity require another person’s involvement. A robotic showerhead could therefore contribute to autonomy by increasing control over water positioning, rinsing, and other portions of showering while leaving other forms of assistance unchanged. Such an interpretation avoids assuming that greater automation necessarily produces greater independence. Participants who already showered independently anticipated less benefit, suggesting that the value of the technology depends on whether it addresses a meaningful gap between what an individual wants to do and what they can currently accomplish.

This task-specific framing extended prior findings on older adults’ preferences for robot assistance. Smarr et al. [37] found that older adults were selective about the everyday activities for which they wanted robot assistance and generally preferred human assistance for personal-care activities. The present findings suggested that even within an intimate activity such as showering, robotic assistance may nevertheless be acceptable when it is applied selectively to particular components of the activity that increase users’ control and reduce unwanted hands-on assistance.

SMEs highlighted the need to balance independence with safety. Care partners may remain concerned about falls, misuse, or the user attempting tasks beyond their current abilities. Assistive robotic systems for activities of daily living may benefit from being designed around graduated assistance rather than an all-or-nothing model of independence. Users may need different levels of support across tasks and over time as their abilities change. Features such as an accessible emergency control, caregiver override, optional status alerts, movement limits, and safe preset routines may allow the user to retain control while maintaining appropriate safeguards. A system that can provide assistance only when needed may therefore better preserve agency than one that automatically takes control of the entire activity.

### 4.2. Functional Fit Matters More than Designing for a Single Disability Profile

Participants’ responses across the concepts and interaction modes consistently emphasized variability in strength, reach, speech, involuntary movement, cognition, and other functional abilities. Importantly, participants did not assume that people with the same diagnosis would interact with the system in the same way. Diagnosis or disability may be an insufficient basis for determining how an assistive robotic system should move or how users should control it. This finding closely reflects the principles of ability-based design, which argued for designing accessible systems around what users can do, that is, creating systems that bends to fit users’ abilities rather than centering on overall disability categories [38]. The present findings extended this perspective to robotic showering by demonstrating that relevant abilities encompass not only physical reach and strength but also speech, movement control, cognition, and preferred forms of interaction.

The interaction-mode findings illustrate this clearly. Voice control was assumed to reduce physical demands for some participants but could create barriers for individuals with speech or communication limitations. Gesture interaction could provide an alternative for some users yet could be difficult for individuals with restricted upper-extremity movement or involuntary movements. Manual control was viewed as valuable because of its familiarity and directness but perhaps not feasible for every user.

The emerging interest in combinations of user-guided and voice control therefore appears less important as a preference hierarchy than as evidence that participants valued the ability to match interaction to their own capabilities and to the demands of a particular showering task. Related research on voice assistants with individuals with motor, linguistic, and cognitive impairments similarly suggests that speech-based interaction can reduce the need to physically manipulate devices while its accessibility remains contingent on users’ linguistic and cognitive abilities [39]. These findings reinforced why voice interaction may be most useful as one accessible option rather than as a universal replacement for direct manual control.

These results have implications for multimodal design: providing more modalities is not necessarily equivalent to providing greater accessibility. A system containing voice, gesture, manual, and other controls could itself become burdensome if users must understand or manage all of them. Instead, multimodality may be most useful when modes can be selectively enabled, simplified, personalized, or ignored. Users should not have to adapt themselves to the complete interaction repertoire of the robot.

The same principle applies to the physical configuration of the showerhead. Participants did not converge entirely around one concept. Although the Elongation concept was frequently viewed favorably, the Swing concept offered advantages in directional reach, and two SMEs preferred a combination of Swing and Elongation characteristics. This suggests that the design space may be better understood in terms of desired capabilities, such as extending toward the user, reaching around the body, remaining where positioned, and retracting when not needed, rather than treating the three concepts as mutually exclusive final designs. Future iterations may therefore benefit from selectively combining the most valued movement characteristics while avoiding unnecessary mechanical complexity.

### 4.3. Familiarity Can Provide an Anchor for Robotic Assistance

Concept preferences suggested that additional robotic capability was valued when it extended familiar showering interactions rather than fundamentally replacing them. The favorable response to the Elongation concept is particularly informative in this regard. Its front-facing orientation resembled conventional showerhead placement, while the robotic functionality expanded reach and positioning. Similarly, user-guided interaction provided a familiar reference point even when participants valued voice control.

This suggests a broader design opportunity for domestic assistive robotics: rather than requiring users to adopt an entirely new interaction model, robotic functionality can be embedded within recognizable household objects and routines. For showering, familiarity may reduce the conceptual distance between a conventional fixture and an assistive robotic device. This may be particularly valuable when a technology is introduced into an activity that users have performed for decades and for which they have established personal routines and preferences.

At the same time, familiarity should not be interpreted as resistance to innovation. Participants expressed interest in voice control, automated positioning, personalized settings, and combinations of movement mechanisms. Their responses suggested that unfamiliar capability may be easier to accept when layered onto an understandable baseline. The design challenge is therefore not simply to maximize robotic functionality but to determine how much additional capability produces meaningful benefit without making the fixture unnecessarily difficult to understand, manipulate, maintain, or install.

This balance was visible in participants’ responses to the Two Showerhead concept. Additional hardware and coverage did not automatically translate into greater perceived value. For some participants, similar functionality appeared achievable through a single sufficiently flexible showerhead. This indicates that technological sophistication should be justified by a distinct functional advantage rather than treated as inherently desirable.

### 4.4. Learnability, Predictability, and Control Are Part of Accessibility

The findings broadened accessibility issues beyond whether users are physically capable of interacting with the showerhead. Participants considered whether they could remember commands, understand directional mappings, distinguish available functions, anticipate how the showerhead would respond, and develop confidence through practice. These issues are particularly relevant in showering because interaction occurs while users may simultaneously be managing balance, washing, handling personal care products, and dealing with other physical demands.

Manual or user-guided control remained important because of familiarity, predictability, and directness. The greater concern surrounding gesture interaction illustrated this relationship between physical and cognitive accessibility. Even when a user could physically perform a gesture, they might still need to remember its meaning or perform it in a sufficiently recognizable way. Similarly, voice interaction may require users to learn particular terminology or understand how the system interprets spatial commands. Accessibility therefore depends on the correspondence between the user’s intended action and the system’s interpretation, not simply on whether an input can technically be produced. These observations support keeping interaction vocabularies limited and predictable and maintaining a familiar method of direct control when possible.

The results suggested value in gradual familiarization. Rather than exposing users to every available function at once, basic interaction could be established first, with additional modes or automated functions introduced as users become comfortable with the system. For individuals whose abilities change over time, this approach may also allow for interaction strategies to evolve without requiring them to learn an entirely new system. Participants’ comments also suggest value in becoming familiar with assistive technologies before they become essential for everyday functioning. Early exposure may allow users to build confidence, learn interaction strategies, and establish routines while they still have greater functional capacity, potentially making later transitions to increased reliance on assistive support less disruptive.

Brief exposure to a domestic assistive robot has been shown to increase older adults’ perceptions of usefulness and ease of use [40], whereas a one-month Living Lab study found that learning and retention demands differed across users and that direct experience did not necessarily translate into greater intention to use the robot [41]. Together, these findings reinforced the importance of examining learnability and sustained value rather than relying only on initial impressions.

The findings revealed a potential tension between adaptability and cognitive load. Multiple modes can make the system accessible to a wider range of users, but too many commands, gestures, showerhead positions, and settings may become difficult to remember or manage. The present study did not directly assess how difficult these modes would be to learn because participants evaluated animated concepts rather than operating a functional system. Learning demands should be examined empirically, through exploration of initial preferences as well as command errors, recovery from mistakes, learning across repeated use, and whether preferences change after participants gain direct experience with each interaction mode.

### 4.5. A Robotic Showerhead Must Fit the Household, Not Just the User

Participants evaluated the showerhead within the broader context of the bathroom rather than as an isolated interface. This highlighted a particularly important characteristic of assistive technology intended for the home: successful adoption depends not only on the interaction between one user and one device but also on how the technology fits the physical, social, and infrastructural environment in which it will remain. This contextual perspective is consistent with person–environment research emphasizing that everyday functioning is influenced by the fit between individual capabilities and characteristics of the home environment [36], as well as technology-acceptance research showing that adoption is shaped by contextual factors beyond interface usability alone [42].

The shared nature of bathrooms illustrates this issue. A showerhead designed around one person’s accessibility requirements could create inconvenience for a spouse, family member, or guest who uses the same shower differently. Personalization therefore has a household-level dimension. Saved settings, retractability, adjustable positioning, and continued conventional operation may allow accessibility features to coexist with ordinary use rather than converting the bathroom into a space optimized exclusively for one individual.

Furthermore, aesthetic integration may influence whether the system is viewed as a desirable home product rather than specialized medical equipment. A bulky, highly technological, or institutional appearance could discourage adoption, create stigma, or make other household members less willing to use the same shower. The concern about an institutional appearance is consistent with broader assistive-technology literature identifying stigma as a recurring barrier to adoption [43]. The present findings extend this concern beyond the individual user by showing that the appearance and operation of an assistive device may also matter because it occupies a shared household space used by people with different accessibility needs.

Privacy concerns reinforced the importance of considering the character of the activity itself. Participants’ strong resistance to cameras suggested that sensing approaches acceptable elsewhere in the home may not be acceptable during showering. In this context, adding sensing simply because it improves technical capability could undermine acceptance of the entire system. The findings therefore favored a principle of sensing minimality: collect only the information necessary to provide the intended functionality and, where feasible, favor less visually identifiable forms of sensing. Users should be able to understand when sensing is active and what role it plays in system operation. Privacy concerns surrounding sensing technologies have similarly been reported in prior studies with older adults [23]. The strong rejection of cameras in the present study suggests that such concerns may become particularly consequential when sensing is embedded within an intimate activity such as showering.

The same contextual perspective applies to installation and affordability. Retrofit ability may be as consequential as interface usability. Plumbing requirements, housing permissions, water containment, maintenance, and cost were not peripheral concerns raised after participants evaluated usability; they influenced how participants judged the concepts themselves. This suggests that implementation feasibility should be treated as part of the design problem from the beginning. Future prototypes should be evaluated in realistic environments. A highly accessible interaction may have little practical value if the system requires extensive renovation, cannot be installed in rental or senior-living environments, creates water-management problems, or is unaffordable.

### 4.6. Implications for Future Soft Robotic Showerhead Design

Across these broader findings, several design directions emerged. First, future research should test functional prototypes in realistic seated-showering scenarios. Concept interviews reveal expectations and concerns, but hands-on use is necessary to determine whether participants can reach, reposition, command, stop, and recover the system safely. Findings should be interpreted as perceptions of anticipated usability, acceptability, and potential benefit. Future prototype-based and in situ evaluations are therefore needed to determine whether these perceived benefits translate into measurable improvements in showering performance, safety, and independence.

Second, the physical and interaction design should be configurable around functional ability. Studies should recruit participants with a wide range of functional profiles rather than treating age or diagnosis as proxies for ability. Researchers should evaluate hybrid control configurations rather than comparing concepts and modes only in isolation. Studies could examine whether personalized command sets improve performance, and whether reducing the number of active features lowers cognitive burden while addressing need.

Third, personalization should extend beyond water preferences to how the system moves and is controlled. Saved positions, interaction configurations, or household profiles could reduce repeated setup while allowing multiple users to share the same shower. Such personalization should remain simple enough that configuration itself does not become another accessibility barrier.

Fourth, longitudinal research is needed to understand adoption beyond initial usability [41]. Participants may respond positively during a demonstration but discontinue use if the system is difficult to clean, frequently misinterprets commands, interferes with other household members, or requires costly maintenance. Home trials could assess sustained use, changes in showering frequency, caregiver involvement, privacy, confidence, and perceived independence.

Finally, automation should remain transparent and recoverable. Failure modes should default to a safe state, and manual operation should remain available whenever feasible. These characteristics may be particularly important in a wet environment where unexpected movement or misdirected water could create frustration or perceived safety concerns.

Finally, economic and implementation research should compare the cost of the robotic showerhead with caregiver assistance, bathroom renovation, and other assistive bathing solutions. Demonstrating that the system can extend independent bathing, reduce caregiver involvement, or delay more costly support may be important for future reimbursement and adoption.

## 5. Limitations

The findings should be interpreted considering several limitations. Participants responded primarily to video-based animations of proposed concepts rather than prolonged use of a fully installed system in their homes. Their comments therefore reflected anticipated rather than observed usability. The findings should be treated as formative evidence for prototype design and require validation through hands-on testing. The sample included a limited number of older adults and SMEs and was not intended to represent the full range of experiences among older adults with mobility disabilities or individuals who provide bathing assistance. It may not represent the full diversity of mobility, sensory, cognitive, communication, cultural, and housing experiences among potential users. Furthermore, there could be additional identified perspectives or concerns not represented in the present analysis. We therefore interpret the findings as exploratory insights that identify salient design considerations rather than as an exhaustive account of user needs. Nevertheless, the study provided focused, design-relevant insights from both end users and caregiving stakeholders, offering a valuable foundation for refining assistive showering technologies and guiding future usability testing with broader and more diverse populations across a broader range of functional abilities and living environments.

## 6. Conclusions

This study provided formative insight into how older adults who use wheeled mobility devices and shower while seated, together with caregiving stakeholders, envision the role of a soft robotic showerhead in supporting showering. The findings suggested that the value of such a system lies not simply in automating showerhead movement but in enabling assistance that remains controllable, adaptable to individual abilities, compatible with familiar routines, and feasible within real household environments. The results underscored that accessibility cannot be separated from learnability, privacy, shared household use, installation, affordability, and aesthetic needs. Rather than identifying a single optimal concept or interaction mode, the study highlighted the importance of flexible designs that can accommodate differences in functional ability and changing support needs.

These findings offer a foundation for developing and evaluating functional soft robotic showerhead prototypes. Whether the anticipated benefits translate into safer, more private, or more independent showering remains to be established through hands-on and extended evaluations. By designing around individual abilities, changing needs, shared household use, and the realities of existing bathrooms, robotic showering systems may provide meaningful support for independence and preservation of dignity, control, and quality of life.

## Figures and Tables

**Figure 1:**
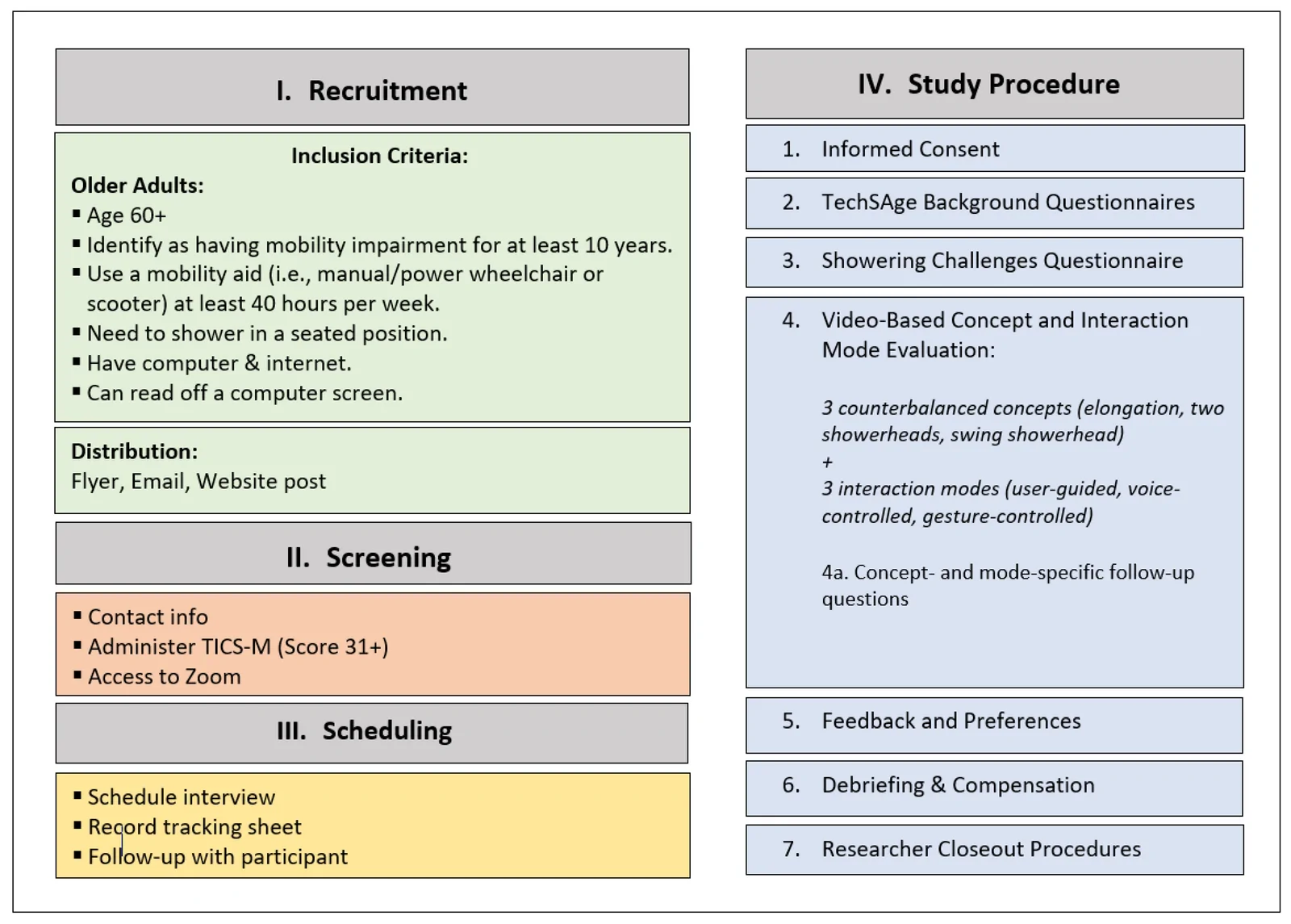
Study flow diagram for older adult participants.

**Figure 2:**
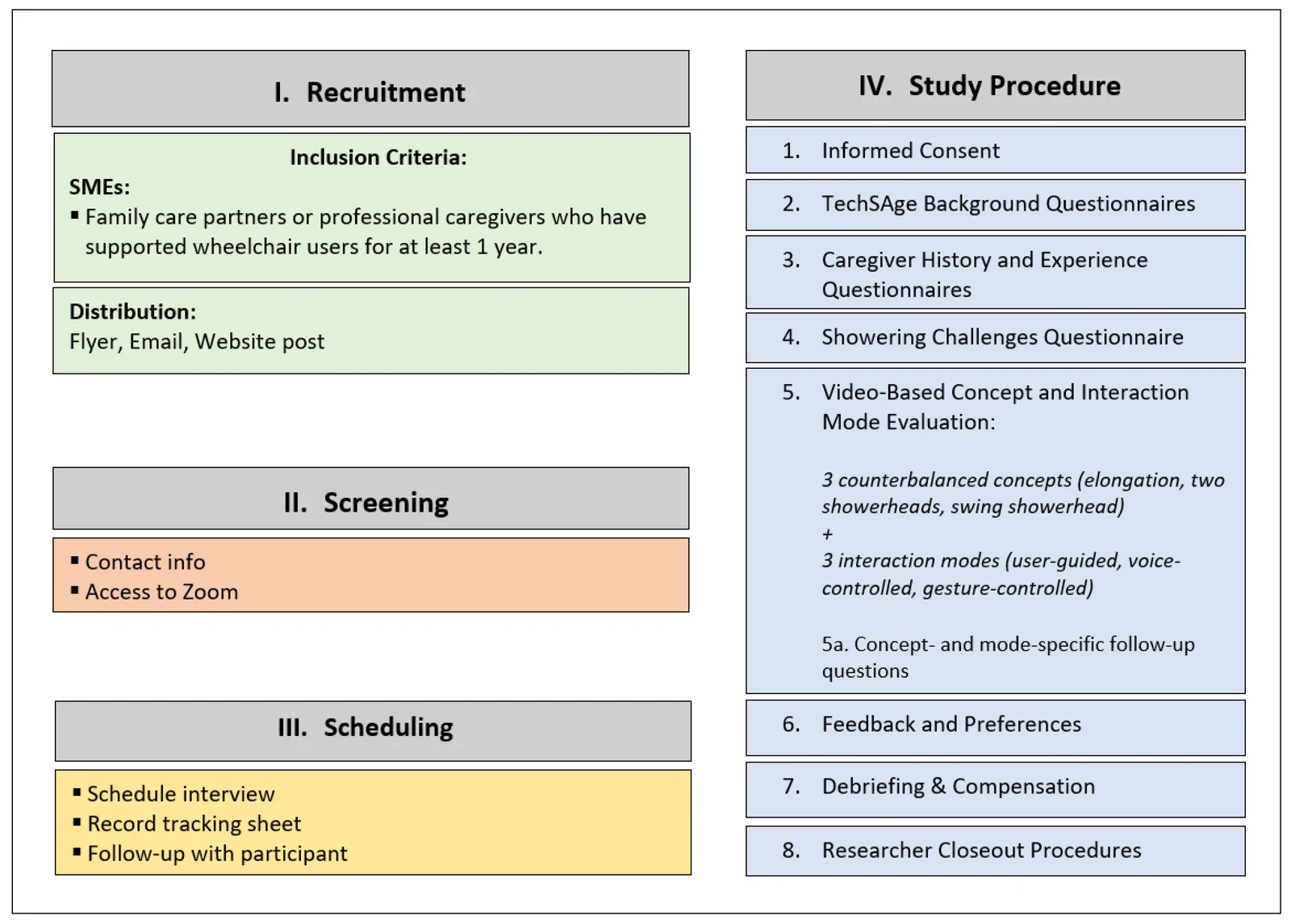
Study flow diagram for subject matter experts (SMEs).

**Figure 3:**
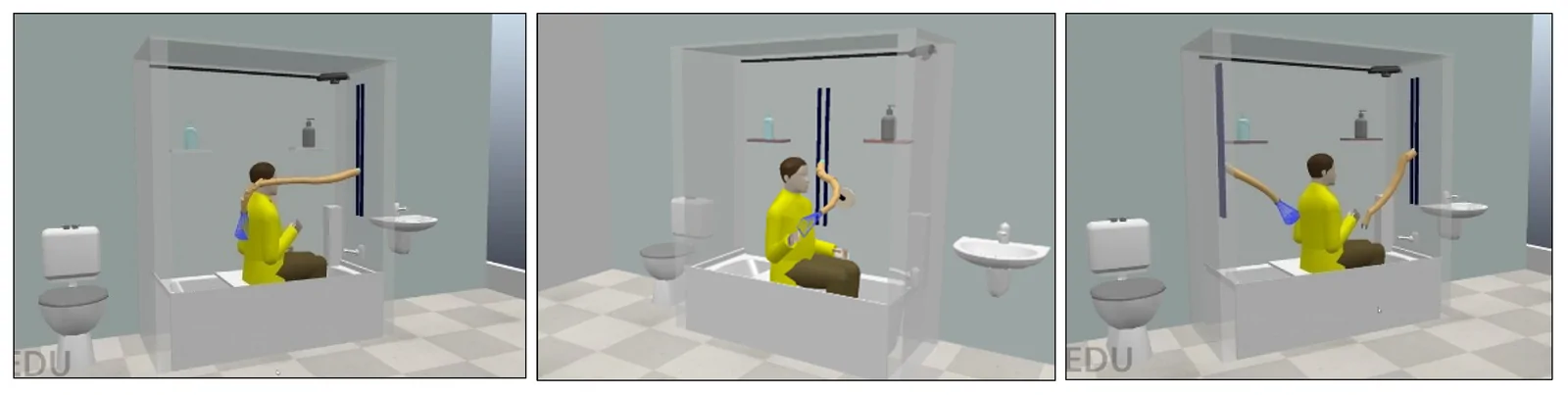
From (**left**) to (**right**) elongation showerhead concept; swing showerhead concept; two showerhead concept.

**Table 1:** Demographic characteristics of older adult participants.

Participant ID	Age	Gender	Race	Disability Status	Duration of Disability
R3_OA_P1_01	77	Male	White	Post-polio syndrome	75 years
R3_OA_P2_02	63	Female	White	Ehlers–Danlos syndrome	15 years
R3_OA_P3_03	76	Female	White	Post-polio syndrome	20 years
R3_OA_P4_04	74	Female	Black or African American	Post-polio syndrome	68 years
R3_OA_P5_05	73	Female	White	Multiple sclerosis	30 years
R3_OA_P6_06	74	Male	White	Post-polio syndrome	>30 years

**Table 2:** Demographic characteristics of subject matter expert participants.

Participant ID	Age	Gender	Race	Caregiver Role	Duration of Caregiver Role
R3_SME_P2_02	78	Female	White	Professional and Personal	~30 years
R3_SME_P3_03	65	Female	White	Personal	15 years
R3_SME_P4_04	69	Female	White	Personal	8 years
R3_SME_P5_05	30	Female	Multiracial	Personal	3 years
R3_SME_P6_06	68	Female	Multiracial	Professional and Personal	11 years
R3_SME_P1_07	55	Female	White	Personal	5 years

**Table 3:** Counterbalancing protocols for presentation of the three robotic showerhead concepts.

Protocol	Concept Order
P1	Elongation → Two → Swing
P2	Elongation → Swing → Two
P3	Two → Elongation → Swing
P4	Two → Swing → Elongation
P5	Swing → Elongation → Two
P6	Swing → Two → Elongation

Note. Each protocol represents a unique sequence for presenting the three concepts. The same counterbalancing structure was applied across older adult and SME sessions, while the interaction-mode sequence within each concept remained fixed.

**Table 4:** Concept and mode preferences of participants.

Participant ID	Concepts(s) and Mode(s) Preferences of Participants	
	Concept(s)	Mode(s)
R3_OA_P1_01	Swing	Voice-controlled
R3_OA_P2_02	Elongation	User-guided
R3_OA_P3_03	Elongation	User-guided
R3_OA_P4_04	Elongation	User-guided with Voice-controlled
R3_OA_P5_05	Elongation	User-guided with Voice-controlled
R3_OA_P6_06	Two Showerhead	User-guided with Voice-controlled
R3_SME_P2_02	Elongation	Voice-controlled
R3_SME_P3_03	Elongation	User-guided with Voice-controlled
R3_SME_P4_04	Swing with Elongation	User-guided
R3_SME_P5_05	Swing with Elongation	User-guided with Voice-controlled
R3_SME_P6_06	Elongation	Voice-controlled
R3_SME_P1_07	Elongation	User-guided with Voice-controlled

## Data Availability

Further inquiries can be directed to the corresponding author.
